# Comparison of High-Risk Breast Cancer Screenings with Concurrent Versus Staggered Breast MRI and Mammogram Schedules

**DOI:** 10.3390/jcm15114239

**Published:** 2026-05-30

**Authors:** Lauren Foster Cornell, Marie Plante, Tanmayi Pai, Zhuo Li, Santo Maimone, Andrey Morozov, Robert Maxwell, Pooja Advani, Kristin Robinson

**Affiliations:** 1 Department of Breast Surgery, Diagnostic Breast Clinic, AdventHealth Cancer Institute, Orlando, FL 32804, USA; 2 Department of Medicine, Mayo Clinic, Jacksonville, FL 32224, USA; 3 Department of Hematology and Oncology, Mayo Clinic, Jacksonville, FL 32224, USAadvani.pooja@mayo.edu (P.A.); 4 Department of Quantitative Health Science, Mayo Clinic, Jacksonville, FL 32224, USA; 5 Department of Radiology, Mayo Clinic, Jacksonville, FL 32224, USA; maimone.santo@mayo.edu (S.M.); morozov.andrey@mayo.edu (A.M.); robinson.kristin@mayo.edu (K.R.)

**Keywords:** breast cancer, breast cancer screening, breast MRI, breast imaging

## Abstract

**Background:** The current guidelines recommend that women at elevated risk for breast cancer (BC), defined as lifetime BC risk ≥ 20%, undergo annual screening with breast magnetic resonance imaging (MRI) and mammogram. However, limited evidence exists in the literature to guide the optimal timing of the MRI relative to the mammogram. Our study evaluated women undergoing high-risk BC screenings to determine the impact of timing for supplemental MRI on BC detection. **Methods:** Patients who completed high-risk screening MRI at a single institution from January 2019 through June 2022 were included. Baseline characteristics and clinical outcomes were collected through retrospective chart review. MRI exams were divided into two groups based on timing of MRI: concurrent (<90 days from mammogram) and staggered (≥90 days from mammogram). **Results:** Of 1115 patients initially identified, 770 met inclusion criteria, with a total of 3707 screening exams performed (2073 mammograms and 1634 MRIs). The final analysis included 1355 MRI exams for 712 patients, where a prior mammogram and MRI were available. Of the MRIs included, 784 (57.9%) were concurrent and 571 (42.1%) were staggered. Additional imaging was performed for 12.5% (98/784) of concurrent MRIs and 9.6% (55/571) of staggered MRIs. Biopsy rates were 9.8% (77/784) for concurrent and 7.0% (40/571) for staggered MRIs. In this high-risk population, there were surprisingly low BC detection rates, with a BC incremental detection rate of 0.5% in both the concurrent and staggered groups (*p* = 1.0), with the median size of detected invasive BCs equaling 5 mm in the concurrent group and 4 mm in the staggered group (*p* = 0.72). **Conclusions:** When comparing concurrent and staggered MRI regimens, there were no significant differences in incremental cancer detection rate, tumor size, rates of additional imaging, or negative biopsies. Further investigation with prospective analysis is needed to validate these findings.

## 1. Introduction

Breast cancer (BC) is the most common non-dermatologic cancer of women in the United States, with an average lifetime risk of developing invasive BC of 13% or 1 out of every 8 women [1]. Many factors influence individual risk for BC including family history, reproductive history, hormone exposures, previous biopsies, mammographic breast density, history of chest wall radiation, and lifestyle behaviors [2]. Patients with hereditary predispositions to breast cancer, including pathogenic variants in *BRCA-1/2* can have a lifetime risk as high as 80% [3,4,5]. Due to advancements in screening technologies and treatment strategies, BC mortality rates have fallen by between 1.8% and 3.4% per year since 1990 [6]. It has been estimated that up to 614,500 BC deaths were averted from 1989 through 2018 due to these advancements [6].

Digital mammography is generally regarded as the preferred initial screening tool for breast cancer, recognizing its’ ability to reduce breast cancer-related mortality [7,8,9]. Still, mammography has limitations, in particular in high-risk women and those with increased mammographic density. The addition of contrast-enhanced breast magnetic resonance imaging (MRI) has been shown to improve cancer detection rates, yielding an additional 8 to 15 cancers per 1000 women screened compared with mammogram alone [10,11,12]. The use of gadolinium-containing contrast agents with MRI allows for assessment of functional changes to the tissue vascularity and early identification of neo-angiogenesis [13]. While many have argued that there is limited added benefit of a screening mammography in patients undergoing breast MRI, the literature has demonstrated benefit in select high-risk patients [14]. One patient data meta-analysis in *BRCA-1/2* carriers found that, in young (<40 years) *BRCA-2* carriers, up to one third of cancers were visualized by mammogram only and missed by MRI [15]. Acknowledging the potential benefit, along with the limited harm of mammography, it is generally accepted that optimal BC screening in high-risk women should include both mammography and breast MRI [2,14,16]. The current American Cancer Society guidelines recommend that women with a lifetime risk of 20% or greater based on validated risk assessment tools such as Claus, Tyrer–Cuzick and BRCAPRO models undergo annual breast MRI in addition to yearly mammograms [2]. This recommendation for supplemental MRI also applies to those patients with hereditary BC syndromes (such as *BRCA-1/2*), which increase remaining BC risk above 20% and those with a history of high-risk intraepithelial lesions, such as atypical hyperplasia (AH) or lobular carcinoma in situ (LCIS), which are known to substantially increase future breast cancer risk above this threshold [2,3,14,17].

Despite evidence and guidelines recommending use of supplemental breast MRI in conjunction with mammography to screen high-risk patients, there is no clear consensus regarding ideal timing for supplemental MRI relative to the mammogram. There is theoretical benefit to performing annual mammography and breast MRI at the same time or “concurrent,” as this could potentially allow immediate comparisons between the two imaging modalities for better overall evaluation of the breasts. On the contrary, more-frequent screening imaging with “staggered” breast MRI and mammography six months apart could potentially expedite cancer detection and enable detection of more interval BCs [18]. In one retrospective study of 73 *BRCA-1/2* patients undergoing screening MRI and staggered mammography, MRI detected 12 cancers that were not visualized by mammogram 6 months prior. However, 5 of the 12 cancers were found on an initial MRI, suggesting a component of prevalent cancers, which may have been mammographically occult [19].

Acknowledging the gap in the available literature, the current study aimed to compare the effectiveness of concurrent verses staggered screening schedules in high-risk women, specifically with regards to incremental cancer detection rates. Secondary objectives also evaluated differences in the size of screen-detected cancers and rates of false-positive findings.

## 2. Methods

**Figure 1:**
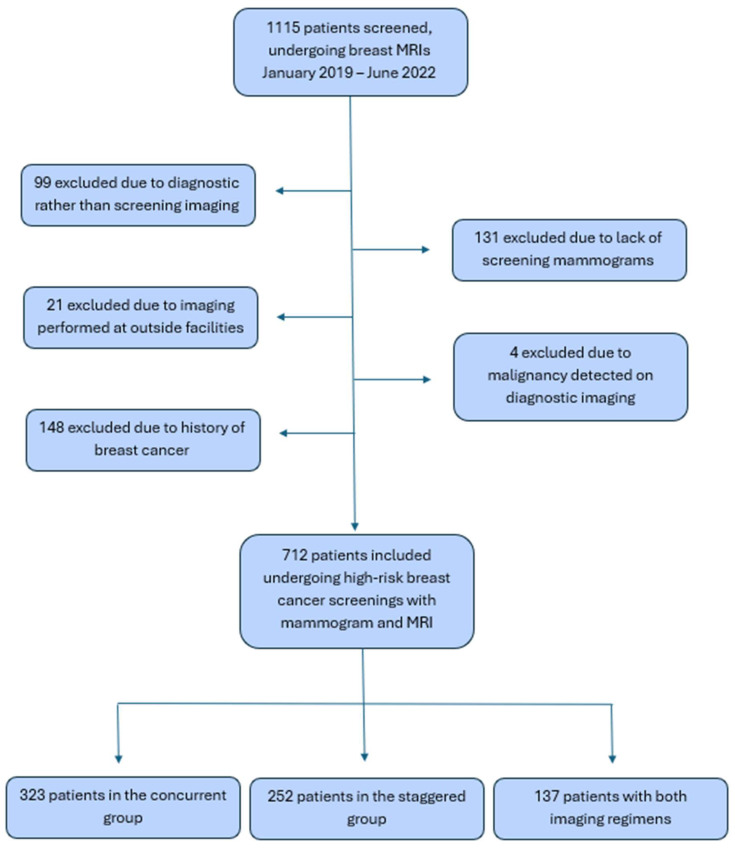
Inclusion and exclusion flow chart.

This retrospective, observational study was performed at a single center in the United States. This study underwent review and approval by the Institutional Review Board (IRB 22-002115). Data were de-identified to all but the study investigators. Females 30 years and older who met guideline-based criteria for and completed high-risk annual BC screening with digital mammography and supplemental breast MRI from January 2019 through June 2022 were included. Patients were identified through a radiology record database. Patients with a history of breast cancer, those presenting for diagnostic imaging or with imaging performed at outside institutions, and those without prior negative prevalence screening MRI were excluded. All imaging analyzed had either cancer diagnosis or 12-month follow-up with no cancer. See summary in Figure 1. Baseline demographics, estimated risk of BC (as calculated via the validated Tyrer–Cuzick model), presence of pathogenic hereditary BC gene variants, history of high-risk intraepithelial breast lesions, and prior use of BC prevention medications were collected for each patient. Imaging date, breast imaging reporting and data system (BI-RADS) assessment, breast density pattern, background parenchymal enhancement (BPE), hormone status at time of imaging, additional diagnostic imaging, and the number of biopsies performed were collected for each screening exam. If a biopsy was performed, the results were documented as benign, malignant, or high risk (defined as either atypical lobular hyperplasia [ALH], atypical ductal hyperplasia [ADH] or LCIS). All MRIs were performed using a 1.5 Tesla magnet strength and included administration of gadolinium-containing contrast agent with Gadavist^®^ (gadobutrol). Characteristics documented for detected malignancies included: pathologic size of surgical specimen, Nottingham grade, ductal or lobular histology, presence of invasive component, estrogen receptor (ER) status, progesterone receptor (PR) status, human epidermal growth factor 2 (HER2) receptor status, and presence of lymph node involvement. MRI exams were divided into two groups based on timing of MRI: concurrent group (<90 days from mammogram) and staggered group (≥90 days from mammogram).

Statistical analysis was completed with categorical variables summarized as frequency (percentage) and continuous variables reported as median (range) and mean (standard deviation). For the exploratory analysis, concurrent and staggered imaging schedules were compared using Wilcoxon rank sum test for continuous variables and Fisher’s exact test for categorical variables, assuming each exam from the same patient was independent of others.

## 3. Results

### 3.1. Study Population

In total 1115 patients who underwent breast MRI were reviewed, with 712 patients included in final data analysis. Over the study period, individual patients may have had both concurrent and staggered MRIs. Therefore, patients were divided into three subgroups: 323 exclusively had concurrent MRIs, 252 exclusively had staggered MRIs, and 137 had a combination of years with concurrent and staggered regimens. Groups were matched for age, ethnicity, estimated Tyrer–Cuzick score, history of high-risk intraepithelial lesions, and use of BC prevention medications. Patients with hereditary gene mutations for BC were more likely to have staggered or combination regimens rather than concurrent regimens (*p* = 0.007). See Table 1 for a summary of baseline characteristics.

Tyrer–Cuzick score was available for 552 patients with a median score of 26% (range 10.2–74%). A total of 133 patients had a history of high-risk intraepithelial lesions including ADH (n = 70), ALH (n = 67) and LCIS (n = 24), with some patients having more than one type of high-risk intraepithelial lesion. A total of 63 patients were known to carry a pathogenic or likely pathogenic variant in a hereditary BC gene including *BRCA1* (n = 9), *BRCA2* (n = 15), *CHEK2* (n = 16), *PALB2* (n = 6), *ATM* (n = 4), and Other (n = 13).

A total of 84 patients had been prescribed BC prevention medications as follows: tamoxifen (n = 26, 30.9%), raloxifene (n = 24, 28.6%), exemestane (n = 25, 29.7%), and anastrozole (n = 20, 23.8%). Of these patients, 59 (70%) were currently using prevention medications at the time of MRI imaging.

Among the 712 patients included, there were 3707 screening exams (2073 mammograms and 1634 MRIs). Final analysis included 1355 MRI exams of which 784 (57.9%) were concurrent and 571 (42.1%) were staggered with the screening mammogram.

BI-RADS distributions for the concurrent and staggered mammograms and MRIs were similar between groups (*p* = 0.097 and *p* = 0.16, respectively). This is summarized in Table 2.

### 3.2. Clinical Outcomes

Incremental BC detection rates were 0.5% or 5 per 1000 for both the concurrent and staggered MRI groups. False-positive MRI assessments, defined by any MRI with BI-RADS of 0, 3, 4 or 5, where malignancy was not diagnosed, occurred in 182 (23.2%) of 784 concurrent MRI exams and in 115 (20.1%) of 571 staggered MRI exams (*p* = 0.18). Additional imaging was performed for 12.5% (98/784) of concurrent MRI exams and 9.6% (55/571) of staggered MRI exams (*p* = 0.12). A total of 143 biopsies were performed based on MRI findings, with 82 prompted by presence of non-mass enhancement and 61 prompted by mass enhancement. The number of MRIs prompting biopsy occurred at similar rates between groups, 77 of 784 (9.8%) exams for concurrent imaging and 40 of 571 (7.0%) exams for staggered imaging (*p* = 0.08). As some MRIs may have prompted more than one biopsy, a total of 97 biopsies were performed from 77 concurrent MRI exams, and 46 biopsies were performed from 40 staggered MRI exams. This is summarized in Table 3. Of the 97 biopsies performed from concurrent MRIs, 80 (82.5%) were benign, 13 (13.4%) were high risk, and four (4.1%) were malignant. Of the 46 biopsies performed from staggered MRIs, 36 (78.2%) were benign, seven (15.3%) were high risk (ADH, ALH or LCIS), and three (6.5%) were malignant. PPV3 for concurrent MRI exams was 4.1% (4/97) and for staggered MRI exams was 6.5% (3/46). If high-risk biopsies are included, PPV3 increased to 17.5% (17/97) for concurrent exams and 21.7% (10/46) for staggered exams. Comparison of outcomes and biopsy results between MRI schedules is demonstrated in Table 3. No malignancies were detected by screening mammography in this high-risk population undergoing annual MRI.

**Figure 2:**
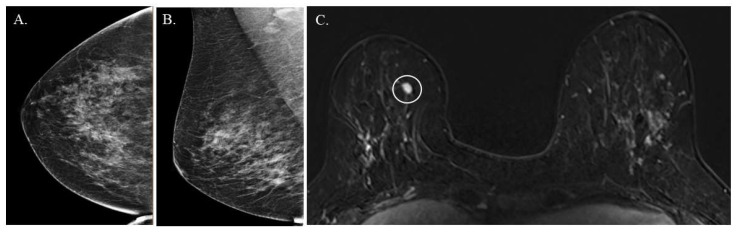
A 50-year-old female with calculated TCv8 lifetime risk of 26.5% undergoing annual mammography and supplemental screening breast MRI in a concurrent regimen. (**A**,**B**) Right breast screening mammogram with craniocaudal (CC) and mediolateral oblique (MLO) views demonstrates category C tissue density (heterogeneously dense) without suspicious abnormality. (**C**) Breast MRI, axial post-contrast, subtraction image of the inner right breast shows a 7 mm irregular enhancing mass and mild background parenchymal enhancement (circle). Biopsy confirmed invasive ductal carcinoma.

**Figure 3:**
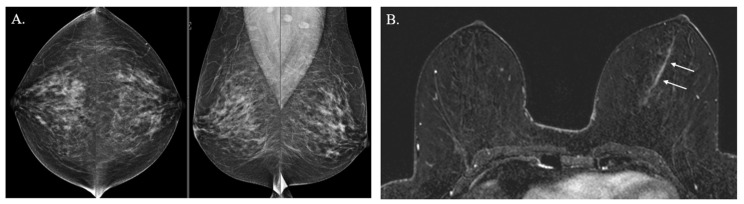
A 53-year-old female with TCv8 lifetime risk of 22.8% secondary to breast density and family history undergoing screening mammography and supplemental screening in a staggered regimen. (**A**) Bilateral screening mammography (CC and MLO views) shows category C density (heterogeneously dense) without mammographic abnormality. (**B**) Breast MRI, axial post-contrast, subtraction image shows 6.4 cm of linear non-mass enhancement in the central left breast (arrows). Biopsy reveals intermediate-grade DCIS with comedo necrosis.

The median size of screen-detected BCs at time of surgical excision was 5 mm in the concurrent group and 4 mm in the staggered group (*p* = 0.72). All patients were pathologically node-negative. Of the five malignancies detected on concurrent MRI, two were invasive ductal carcinoma (IDC), both estrogen receptor and HER2/Neu receptor negative, and three were intermediate to high-grade ductal carcinoma in situ (DCIS). Of the four malignancies detected on staggered MRI two were invasive ductal carcinoma (IDC), both of which were estrogen receptor positive and HER2/Neu receptor positive; one was low-grade DCIS; and one DCIS with microinvasion. No lymphatic or vascular invasion was present within any malignancy detected. See Figure 2 and Figure 3 for images of MRI-detected cancers in both the concurrent and staggered groups.

## 4. Discussion

This study utilized a specialty breast clinic to identify women at high risk for BC undergoing annual mammography with supplemental breast MRI to better understand optimal timing of the screening MRI relative to the annual mammogram. Our results showed that the timing of supplemental screening MRI relative to the screening mammogram did not result in an observed difference in cancer detection rates or size of screen-detected BCs within this high-risk population. The incremental cancer detection rate was overall lower than expected, albeit the same between the concurrent and staggered MRI regimens, at 5 per 1000 exams. The median sizes of screen-detected cancers in both groups were under 1 cm, demonstrating the high sensitivity of screening MRI, regardless of timing. It is notable that the PPV3 was lower than traditional benchmarks for MRI-guided needle biopsies, although the overall biopsy rates were similar to expected ranges. We suspect that the lower PPV3 is due to the exclusion of diagnostic studies, as well as the low overall malignancy rates in the study population. Further studies conducted in a prospective manner will be useful to validate the results and confirm generalizability.

Numerous studies show that earlier detection of BC with screening mammography decreases disease-specific mortality and morbidity [20]. Supplemental screening MRIs within the high-risk population have well-established benefits with increased cancer detection rates, including the detection of interval cancers, and a reduction in late-stage disease [2,10,16,21]. In a retrospective study of asymptomatic women with atypical hyperplasia (AH) or lobular carcinoma in situ (LCIS) undergoing high-risk screening, 4% of patients who had negative mammogram results were found to have a cancer seen only by supplemental MRI [16]. Furthermore, in a study performed in 2014 with 2207 high-risk women who underwent screening with mammography alone, MRI alone, or combined mammogram and MRI, the combined imaging was found to be the most effective in identifying BC at 71% versus 65.7% with MRI alone and zero detected by mammogram alone [14].

A criticism of breast MRI is the burden of false positives, which can contribute to patient anxiety and additional imaging work-ups [2,22]. In our study, similar rates of additional diagnostic imaging were recommended for the concurrent and staggered MRI groups, with 12.5% of concurrent MRIs leading to additional imaging compared with 9.6% of staggered MRIs (*p* = 0.12). The percentage of biopsies performed in the concurrent and staggered groups was also similar (*p* = 0.69), with comparable rates of false-positive findings. In a prior, smaller retrospective study of 143 high-risk women comparing concurrent versus alternating screening MRIs, false-positive rates were higher in the concurrent group at 18.2% compared with the alternating group at 10.2% (*p* = 0.02) [22]. The authors felt this was primarily driven by a higher proportion of BI-RADS 3 findings in the concurrent group. In our study, BI-RADS 3 findings were similar between the two groups. Concordant with our findings, biopsy rates in this smaller study were similar for concurrent and alternating MRIs [22].

More patients in our study participated in concurrent imaging, which may be attributable to practice patterns or patient preferences. One of the most significant limitations to effective cancer screening is patient adherence. Within the general-risk population, the incidence of breast screening compliance has not exceeded 80% since the 1990s, although family history of BC has been shown to improve adherence [23,24]. In a multivariate analysis, time to travel to the nearest imaging center was predictive of non-adherence to screening mammograms [24]. Each additional minute of travel time may decrease the odds of undergoing at least one mammographic examination in the 5 years before cancer diagnosis [24,25]. Within the high-risk population, the incidence of non-adherence to screenings is variable; however, there is an added burden with the incorporation of annual screening MRIs to annual mammograms [26]. It has been demonstrated that the travel distance for breast MRI is much greater when compared with that for mammograms [27]. We surmise that the burden of appointments and travel may be more significant when the frequency of imaging is increased from annual to every 6 months. Due to the retrospective nature of our study design, we were not able to assess adherence in patients who were advised to undergo screening MRI; we only reviewed the outcomes in those women who did complete the testing. Bearing in mind our results showed similar clinical outcomes between the two regimens, it may be reasonable to suggest screening MRIs be concurrent with mammograms for women when time and convenience is a potential barrier to adherence.

Despite the perceived inconvenience, there were twice as many hereditary cancer gene carriers in the staggered group than in the concurrent group, which was a statistically significant difference. This finding suggests patients with the highest risk, as seen within the hereditary cancer gene carriers, may still prefer more-frequent screenings. It also underscores the importance of shared decision-making in establishing a high-risk screening protocol, as patient preferences will likely impact adherence to imaging.

Several limitations of this study are important to acknowledge. In this retrospective study, we focused on differences in screen-detected BCs but were unable to evaluate for differences in interval symptomatic breast cancers, which we recognize as a clinically relevant outcome. Additionally, it is important to note the selection bias involved in our study population, which excluded those with cancers detected at outside institutions and those who may not have completed the recommended MRI screening. It is also notable that the rates of incremental detected BCs with MRI were lower than expected in both the concurrent and staggered groups. We attribute this finding primarily to the fact that our institution is a tertiary referral center, and several patients we reviewed had screen-detected BCs found at their local institutions, which were then excluded from our study. Though the sample size was not small, the number of malignant results was small, limiting power to detect the difference in test outcomes between concurrent versus staggered MRIs. The small number of malignancies also prevented our ability to complete a multivariable model to adjust for baseline confounding factors between the study groups. Furthermore, due to the retrospective nature of the study, patients were not randomized to a specific imaging regimen, so many patients underwent both regimens. This forces us to assume in our statistical analysis that each screening exam is independent from ones in the prior year. Ideally, future prospective data collection with randomization should be done to eliminate this limitation. Finally, our study population was predominantly white (85.3% of overall study population). While race and ethnicity were matched between imaging regimens, we recognize that women of under-represented minorities are more likely to present with more-aggressive molecular subtypes of BC and further studies should be done to establish preferred timing and frequency of high-risk screenings in these populations [28].

In conclusion, this study demonstrates that the timing of supplemental MRI relative to screening mammogram did not lead to any observed difference in cancer detection rates, size of screen-detected BCs, or risk for false-positive findings in women at high risk for BC. Further investigation utilizing larger sample sizes and prospective analysis is needed to validate our findings, and patient-reported surveys as well as physician preferences on concurrent versus staggered imaging schedules may also be included. When counseling patients on the optimal screening schedule, we recommend individualizing the recommendation based on a patient’s unique concerns. Most importantly, we emphasize maintaining regular annual guideline-based screenings for patients who qualify. We recognize the diversity among the high-risk patient population with various levels of social and financial support, which affects the ability to adhere to regular screenings. Concurrent imaging may be more feasible for patients who have less support or have a prolonged commute to imaging centers, while staggered imaging may offer more reassurance to patients with extremely high risk, such as those with hereditary cancer genetic risk. Ultimately, shared decision-making between providers and patients should be utilized to determine the appropriate timing of high-risk screening for an individual patient.

## Figures and Tables

**Table 1:** Demographic and clinical data of the cohort.

	Entire Cohort, N = 712 ^1^			
	Concurrent, N = 323 N (%)	Staggered, N = 252 N (%)	Both, N =137 N (%)	*p* Value
Age, median (range) ^2^	52 years (28–80)	52 years (30–74)	51 years (34–71)	0.27
Race/Ethnicity				
White	279 (86.4)	214 (84.9)	114 (83.2)	0.64
African American	19 (5.9)	20 (7.9)	12 (8.8)	0.43
Asian	16 (5.0)	11 (4.4)	35 (4.9)	0.79
Pacific Islander	8 (2.5)	4 (1.6)	2 (1.5)	0.77
Hispanic	10 (3.1)	10 (4.0)	4 (2.9)	0.86
American Indian	0	2 (0.8)	0	0.16
Other	1 (0.3)	1 (0.4)	1 (0.7)	1.00
Tyrer–Cuzick mean lifetime percentage risk	N = 250, 27.2	N = 192, 28.0	N = 110, 28.0	0.66, 0.80
History of high-risk intraepithelial lesions	69 (21.8)	43 (17.3)	21 (15.4)	0.22
Types of high-risk intraepithelial lesions present				
Atypical ductal hyperplasia (ADH)	36 (11.1)	22 (8.7)	12 (8.8)	0.59
Atypical lobular hyperplasia (ALH)	36 (11.1)	24 (9.5)	7 (5.1)	0.12
Lobular carcinoma in situ (LCIS)	12 (3.7)	6 (2.4)	6 (4.4)	0.52
Presence of hereditary cancer genes	17 (5.3)	30 (11.9)	16 (11.7)	0.007
Hereditary breast cancer genes present				
*BRCA1*	3 (17.6)	4 (13.3)	2 (12.5)	0.95
*BRCA2*	6 (35.3)	6 (20.0)	3 (18.8)	
*CHEK2*	3 (17.6)	9 (30.0)	4 (25.0)	
*ATM*	1 (5.9)	2 (6.7)	1 (6.2)	
*PALB2*	1 (5.9)	2 (6.7)	3 (18.8)	
Other ^3^	3 (17.6)	7 (23.3)	3 (18.8)	0.096
Any use of breast cancer prevention medications	39 (12.1)	26 (10.3)	19 (13.9)	0.56
Current use of prevention medications	23 (59.0)	20 (76.9)	16 (84.2)	0.11
Last year of prevention medication use, median (range)	2019 (2004–2022)	2017 (2005–2022)	2014 (2012–2021)	0.78
Prevention medications used				
Tamoxifen	14 (4.3)	8 (3.2)	4 (2.9)	0.75
Raloxifene	10 (3.1)	7 (2.8)	7 (5.1)	0.46
Exemestane	11 (3.4)	7 (2.8)	7 (5.1)	0.49
Anastrozole	8 (2.5)	9 (3.6)	3 (2.2)	0.70

^1^ Median (range) is reported for continuous variables, and N(%) is reported for categorical variables.^2^ Median age at first mammogram exam collected. ^3^ Other types of hereditary breast cancer genes represented include: APC, BRIP1, CDH1, Lynch syndrome, MUTYH, Peutz–Jeghers syndrome, PTEN, NBN, or multiple genes present.

**Table 2:** Distribution of BI-RADS scores for concurrent versus staggered MRI and mammogram examinations.

MRI Exams				
	Concurrent (N = 784)	Staggered (N = 571)	Total (N = 1355)	*p* Value
**BI-RADS score**				0.16
0	41 (5.2%)	26 (4.6%)	67 (4.9%)	
1/2	602 (76.8%)	456 (79.9%)	1058 (78.1%)	
3	60(7.7%)	51 (8.9%)	111 (8.2%)	
4/5	81 (10.3%)	38 (6.7%)	119 (8.8%)	
**Mammogram Exams**				
	**Concurrent (N = 780)**	**Staggered (N = 516)**	**Total (N = 1296)**	***p* Value**
**BI-RADS score**				0.097
0	77 (9.9%)	49 (9.5%)	126 (9.7%)	
1	260 (33.3%)	192 (37.2%)	452 (34.9%)	
2	417 (53.5%)	268 (51.9%)	685 (52.9%)	
3	21 (2.7%)	4 (0.8%)	25 (1.9%)	
4	5 (0.6%)	3 (0.6%)	8 (0.6%)	

Median (range) is reported for continuous variables, and N (%) is reported for categorical variables.

**Table 3:** Test results by concurrent versus staggered MRI exams.

Total MRI Exams (N = 1355) ^1^			
	Concurrent, N = 784 N (%, 95%CI)	Staggered, N = 571 N (%, 95%CI)	*p* Value
Additional imaging performed	98 (12.5, 10.3–15.0)	55 (9.6, 7.3–12.4)	0.12
MRI prompting biopsy	77 (9.8, 7.8–12.1)	40 (7.0, 5.1–9.4)	0.08
Total biopsies performed	97	46	
Biopsies done per MRI			0.14
1	60 (77.9, 67.0–86.6)	36 (90.0, 76.3–97.2)	
2	14 (18.2, 10.3–28.6)	2 (5.0, 0.6–16.9)	
3	3 (3.9, 0.8–11.0)	2 (5.0, 0.6–16.9)	
Malignancies detected	4 (0.5, 0.1–1.3)	3 (0.5, 0.1–1.5)	1.0
Incremental cancer detection rate (CDR)	5/1000	5/1000	
High-risk intraepithelial lesions	13 (1.5)	7 (1.1, 0.4–2.3)	0.48
Median size of cancer detected, mm	5 (3–20)	4 (3–12)	0.72

^1^ Median (range) is reported for continuous variables, and N (%, 95% CI) is reported for categorical variables. 95%CI for the proportion was estimated using the Clopper and Pearson exact binomial test.

## Data Availability

The datasets used and/or analyzed during the current study are available from the corresponding author on reasonable request.
